# DIETARY DIVERSITY AND OVERWEIGHT ARE ASSOCIATED WITH HIGH INTRINSIC CAPACITY AMONG CHINESE URBAN OLDER ADULTS

**DOI:** 10.1093/geroni/igae098.3465

**Published:** 2024-12-31

**Authors:** Siyang Lin, Jingyi Chen, Xinye jiang, Xiaoming Huang, Yin Yuan, Na Li, Feng Huang, Pengli Zhu

**Affiliations:** Shengli Clinical Medical College of Fujian Medical University, Fuzhou, Fujian, China (People’s Republic); Shengli Clinical Medical College of Fujian Medical University, Fuzhou, Fujian, China (People’s Republic); Fujian University of Traditional Chinese Medicine, Fuzhou, Fujian, China (People’s Republic); Shengli Clinical Medical College of Fujian Medical University, Fuzhou, Fujian, China (People’s Republic); Fujian Provincial Hospital, Fuzhou, Fujian, China (People’s Republic); Fujian Provincial Hospital, Fuzhou, Fujian, China (People’s Republic); Fujian Provincial Hospital, Fuzhou, Fujian, China (People’s Republic); The Shengli Clinical Medical College of Fujian Medical University, Fuzhou, Fujian, China (People’s Republic)

## Abstract

**Background and objectives:**

The associations of intrinsic capacity (IC) with dietary diversity and body mass index (BMI) remain unclear in older adults. This study aimed to examine the associations of dietary diversity and BMI on high IC.

**Methods:**

The cross-sectional study used data from the Fujian Prospective Aging Cohort, which included 1972 individuals aged 60-98 from 2020 to 2021. The dietary diversity score (DDS) was constructed with eight food varieties, and consuming ≥five varieties of food daily was considered a high DDS. BMI was grouped into underweight, normal weight, overweight, and obesity according to the Chinese guidelines. High IC was defined as ≥three unimpaired domains of cognition, locomotion, sensory, vitality, and psychology.

**Results:**

Compared with low DDS, high DDS had a positive association with high IC (OR = 1.42, 95 % CI = 1.16-1.74). Compared with normal weight, underweight was inversely related to high IC (OR = 0.18, 95 % CI = 0.09-0.36), overweight was positively related to high IC (OR = 1.65, 95 % CI = 1.33-2.06), while no significant association was observed between obesity and high IC. The restricted cubic spline model exhibited an inverted U-shaped nonlinear curve of BMI and high IC and identified an optimal BMI of 25.7 kg/m2 for high IC.

**Conclusions:**

High DDS is a protective factor of high IC in older adults. Overweight had the most protective association with high IC among the four BMI subgroups. Individuals with overweight and higher dietary diversity had higher IC.

